# Clarification of the status of the type series and of the holotype of Cyclophorus (Glossostylus) koboensis Godwin-Austen, 1915 (Mollusca, Caenogastropoda, Cyclophoridae) in Nantarat et al. (2014)

**DOI:** 10.3897/zookeys.882.38423

**Published:** 2019-10-23

**Authors:** Sheikh Sajan, Basudev Tripathy, Fred Naggs

**Affiliations:** 1 Malacology Division, Zoological Survey of India, Prani Vigyan Bhawan, M-Block, New Alipore, Kolkata 700053, West Bengal, India Zoological Survey of India Kolkata India; 2 Department of Life Sciences, The Natural History Museum, Cromwell Road, London SW7 5BD, UK The Natural History Museum London United Kingdom

**Keywords:** Gastropoda, nomenclature, typification, NZSI, India

## Abstract

Here, the clarification of the “type” status for Cyclophorus (Glossostylus) koboensis Godwin-Austen, 1915 catalogued by Nantarat et al. (2014) is corrected and briefly discussed.

In describing Cyclophorus (Glossostylus) koboensisGodwin-Austen (1915) explicitly stated that his measured specimen from Kobo in the Abor Hills on the right bank of the Tsanspu or Brahmaputra, no. 6015, collected by Kemp, was the type and that it was deposited in the Indian Museum. Lot numbers 6019-20 from Rotung, collected by Kemp, were also deposited in the Indian Museum. All three lots are now held in the National Zoological Collection of Zoological Survey of India (NZSI). Three additional paratype lots, numbers 3579 from Rotung collected by Oakes (Godwin-Austen 1915: figs 4a–d), 3117 from Yamne Valley, and 3045 from Ponging, were deposited in the “BM’’, more correctly the British Museum (Natural History), BM(NH), now the Natural History Museum, London, NHM. Previously included within Assam, these localities now come within the East Siang district of Arunachal Pradesh, India (Table 1). The upper reaches of the Brahmaputra are currently named the Yarlung Tsangpo. During review of the type of *Cyclophorus* and comparing it with the original literature and that of Nantarat et al. (2014), we noticed that the Cyclophorus (Glossostylus) koboensis was erroneously designated as “lectotype” in a recent publication by Nantarat et al. (2014). In this paper, we correct and clarify the type status for Cyclophorus (Glossostylus) koboensis Godwin-Austen, 1915.

**Table 1.** Detailed information on the type series of Cyclophorus (Glossostylus) koboensis Godwin-Austen, 1915 present in the National Zoological Collection of Zoological Survey of India, Kolkata, and Natural History Museum, London.

Type status and registration numbers	Localities in Abor Hills, Arunachal Pradesh	Collector	Latitude / Longitude	Altitude (m.)
**Holotype**	**Type Locality**: Kobo on right bank of Tsanspu or Brahmaputra River	SW Kemp	27.881588, 95.123787	375
NZSI M.6015/1				
**2 Paratypes**	Rotung, East Siang district	SW Kemp	28.133123, 95.14069	506
NZSI M.6019-20				
**4 Paratypes**	Rotung, East Siang district	Oakes	28.133123, 95.14069	506
NHMUK 1903.7.1.3579/1 is Godwin-Austen’s figured specimen Plate XXXVIII, figures 4a-b, and the invalidly designated lectotype of Nantaret et al. 2014: figure 12A (1–3).				
Nanteret et al. (2014) invalidly attributed paralectotype NHMUK 1903.7.1.3579/2, their figure 12B (1–3) = (Godwin-Austen’s figure 4c-d)				
NHMUK 1903.7.1.3579/3–4, unfigured				
**2 Paratypes**	Yamne Valley, East Siang district	SW Kemp (from NHM register)	28.197478, 95.221596	442
NHMUK 1903.7.1.3117/1–2 (register incorrectly states 1 specimen)				
**3 Paratypes**	Ponging, East Siang district	Oakes (from NHM register)	28.18039, 95.202874	700
NHMUK 1903.7.1.3045/1–3				

Godwin-Austen (1915: 495, fig. 4) described and illustrated the “type” specimen of *Cyclophorus koboensis* and clearly stated that “type” specimen was housed in the Indian Museum (= NZSI). However, Nantarat et al. (2014) catalogue of *Cyclophorus* types held in the Natural History Museum, London, failed to recognise the original holotype designation and designated a lectotype for Cyclophorus (Glossostylus) koboensis Godwin-Austen, 1915 (NHMUK 1903.7.1.3579/1). Their lectotype designation is therefore invalid. Nantarat et al. (2014) gave the type locality as Kobo whereas there can be little doubt that the locality for their invalidly designated lectotype was Rotung. This confusion can be attributed to the labelling that accompanies lot 3579 in the NHM collections, which states in Godwin-Austen’s distinctive handwriting on the base of the box containing the four paratypes “*Cyclophorus koboensis*, G-A. Co Type. Kobo R.B. Brahmaputra. Assam. Capt. Oakes R.E.) Rec Ind Mus. Vol. VII. P. 495. Pl XXXVIII. figs 4–4d. 3579.03.VII.1”. ‘Type Indian Museum’ has been subsequently added to the label in a different hand. However, the entry in the registration book gives the locality as ‘Rotung, Abor Hills’; the original description states ‘Rotung (Oakes)’ and this is repeated in the caption to the two paratypes, figures 4a, 4b and 4c, 4d. We conclude that the labelling with lot 3579 was a mistake on Godwin-Austen’s part. The two figured specimens from this lot are shown from different views, two different views for each; these figured paratypes are not labelled separately in the NHM collections but their distinctive markings allow them to be recognised. Inexplicably, the holotype, figure 4, was shown by Godwin-Austen in apertural view only. Figures of standard views of the holotype are provided for the first time (Fig. 1) with detailed information on the type series and the location of collection sites (Table 1).

**Figure 1.**
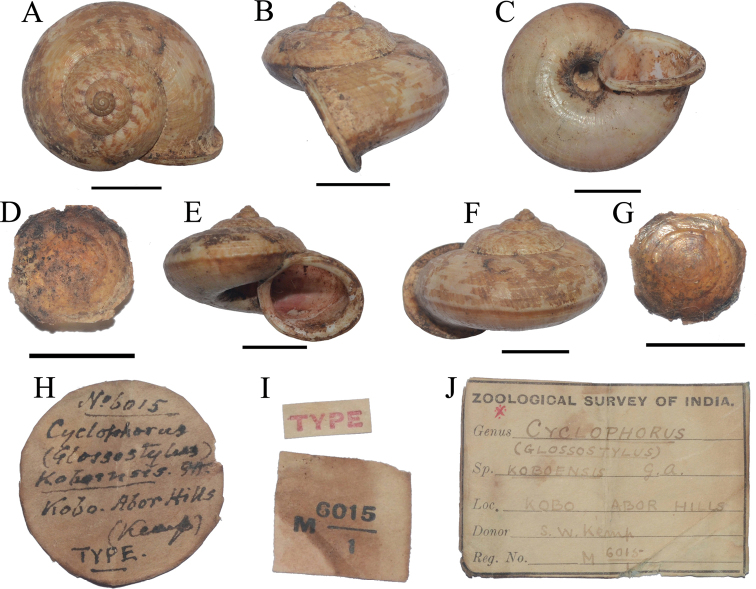
Shell of Cyclophorus (Glossostylus) koboensis Godwin-Austen, 1915 present in National Zoological Collection of ZSI. **A–G** “type” NZSI M.6015/1 (originally designated by author) **H** original handwritten label by Godwin-Austen, 1915 with “type” **I–J** registration number and label. Scale bars: 10 mm.
